# Influence of hyperuricemia treatment on postoperative acute kidney injury among hyperuricemia patients: a single-center retrospective database analysis

**DOI:** 10.1186/s13104-019-4783-1

**Published:** 2019-11-21

**Authors:** Shinichiro Watanabe, Takashi Kawano, Taro Horino, Tatsuki Matsumoto, Keitaro Nagata, Yutaka Hatakeyama, Fabricio M. Locatelli, Masataka Yokoyama, Yoshio Terada, Yoshiyasu Okuhara

**Affiliations:** 1 Center for Innovative and Translational Medicine, Kochi Medical School, Kochi University, Nankoku, Japan; 2 Department of Anesthesiology and Intensive Care Medicine, Kochi Medical School, Kohasu, Oko-cho, Nankoku, Kochi 783-8505 Japan; 3 Department of Endocrinology, Metabolism and Nephrology, Kochi Medical School, Kochi University, Nankoku, Japan; 4 Center of Medical Information Science, Kochi Medical School, Kochi University, Nankoku, Japan

**Keywords:** Acute kidney injury, Allopurinol, Hyperuricemia, Prevention

## Abstract

**Objective:**

Hyperuricemia has been reported to be associated with the development of postoperative acute kidney injury (pAKI). However, it remains underdetermined whether hyperuricemia treatment could decrease the potential risk of pAKI. Here, we investigated this hypothesis among hyperuricemia patients with previously normal renal function by performing a retrospective database analysis.

**Results:**

The study screened 18,169 patients, and were examined preoperative serum creatinine, uric acid, and postoperative serum creatinine. Eight hundred thirty-six patients were finally analyzed for the study, of whom 232 were in the treatment group and 604 were in the non-treatment control group. After adjustment for multi-covariates including baseline (pre-treatment) serum uric acid (SUA) levels, the incidence of pAKI in the treatment group (9.05%; 95% CI 6.04–12.1%) was significantly lower than that in the control group (14.2%; 95% CI 11.2–17.2%). On the other hand, further adjusting for preoperative SUA levels, there was no significant difference in the expected incidence of pAKI between the groups.

## Introduction

Postoperative acute kidney injury (pAKI) is one of the serious complications after major surgery and is associated with significant increases in mortality [1]. However, there is no established therapeutic strategy available [2]. The optimization of the modifiable risk factors for pAKI, i.e., anemia and hypovolemia, is currently considered as the best option for its prevention, while the efficacy is limited [3, 4].

Uric acid is mainly excreted from the proximal tubules as the end product of purine metabolism. Accumulating evidence demonstrates that hyperuricemia is associated with metabolic syndrome, diabetes, hypertension, and chronic kidney disease [5, 6]. Furthermore, recent reports have demonstrated that preoperative hyperuricemia is a potential risk factor for the development of pAKI [7–9]. Meanwhile, it remains underdetermined whether uric acid lowering before surgery could decrease the incidence of pAKI in hyperuricemia patients. Therefore, a prospective placebo-controlled trial for assessment of this hypothesis is required. In this study, we retrospectively investigated the influence of hyperuricemia treatment by allopurinol for pAKI using the propensity score method among hyperuricemia patients with preoperative normal renal function.

## Main text

### Methods

#### Design

A single-center retrospective study was performed at the Kochi Medical School Hospital, which is a 612-bed tertiary care and academic hospital, in Kochi Prefecture, Japan. Anonymized patient data such as basic information, admission and discharge dates, order entry data on laboratory examinations and prescriptions, results of laboratory examinations, physician-registered disease name records, and procedures performed were collected from a physician order entry system and stored in the data warehouse Retrieval sYstem for Open Medical Analysis (RYOMA 2) [10]. All of analyzed data were obtained from the RYOMA 2.

#### Participants

The inclusion criteria were as follows: (1) patients who underwent any type of surgery between January 1, 2000 and December 31, 2016; (2) serum creatinine (SCr) and serum uric acid (SUA) levels were measured within 30 days before surgery; (3) SCr levels were measured within 7 days after surgery; and (4) preoperative estimate glomerular filtration rate (eGFR) was more than 60 mL/min/1.73 m^2^. According to the Japanese guideline for the management of hyperuricemia and gout, hyperuricemia is defined as a SUA level > 7.0 mg/dL (416.4 µmol/L) while the reference ranges for SUA are 3.5 to 7.2 mg/dL (208.2–428.3 μmol/L) and 2.6 to 6.0 mg/dL (154.7–356.9 μmol/L) in males and females, respectively [11]. Patients with preoperative SUA levels > 7.0 mg/dL without treatment were assigned to the control group. On the other hand, patients with baseline (before treatment) SUA levels > 7.0 mg/dL followed by an allopurinol treatment until surgery were classified as the treatment group. In order to minimize the bias regarding surgical-type between groups, the patients that underwent a surgical procedure only presented in the treatment or control group were excluded. Patients who underwent two or more surgeries within 30 days before surgery or 7 days after surgery were also excluded.

#### Study outcome

The primary outcome was the incidence of pAKI. The pAKI was defined according to SCr-based criteria, Kidney Disease: Improving Global Outcomes (KDIGO) [12]: an increase in SCr ≥ 0.3 mg/dL (≥ 26.5 µmol/L) within 48 h after surgery and/or a relative increase ≥ 50% from the preoperative SCr within 7 days after surgery.

#### Propensity score analysis

Propensity scores were estimated for each patient by using boosted classification trees with multi-covariates considered to be related to pAKI [10, 13–15], including baseline SUA levels, i.e., preoperative SUA levels of the control group and SUA levels before allopurinol treatment of the treatment group, age, sex, eGFR, history of urinary lithiasis, hypertension, ischemic heart disease, heart failure, diabetes, cancer and liver disease, prescription of diuretics, angiotensin-converting enzyme (ACE) inhibitors, angiotensin II receptor blockers (ARBs), antibiotics, nonsteroidal anti-inflammatory drugs (NSAIDs), anticancer drugs and contrast media, and type of surgery. The inverse probability treatment weight was calculated from the propensity scores, and used to estimate the average treatment effect. In order to determine if the effect of allopurinol was direct or secondary to SUA lowering, an additional analysis adjusting for preoperative SUA levels instead of baseline SUA levels was further conducted. On the other hand, because of the wide individual distribution with small sample size in each variable, the analysis for determine the effects of allopurinol dosage (Additional file 1: Table S1), as well as treatment length (Additional file 1: Table S2), could not be conducted.

#### Statistical analysis

Normally distributed data was expressed as mean ± standard deviation (SD), whereas percentages were used for categorical variables. An absolute standardized difference less than 0.2 was considered adequate balance between groups [16]. The differences between groups were assessed using the Chi squared test, and the *p* value less than 0.05 was considered statistically significant. All analyses were performed using R statistical software (version 3.0.0, package “twang”; R Foundation for Statistical Computing, Vienna, Austria).

### Results

**Fig. 1:**
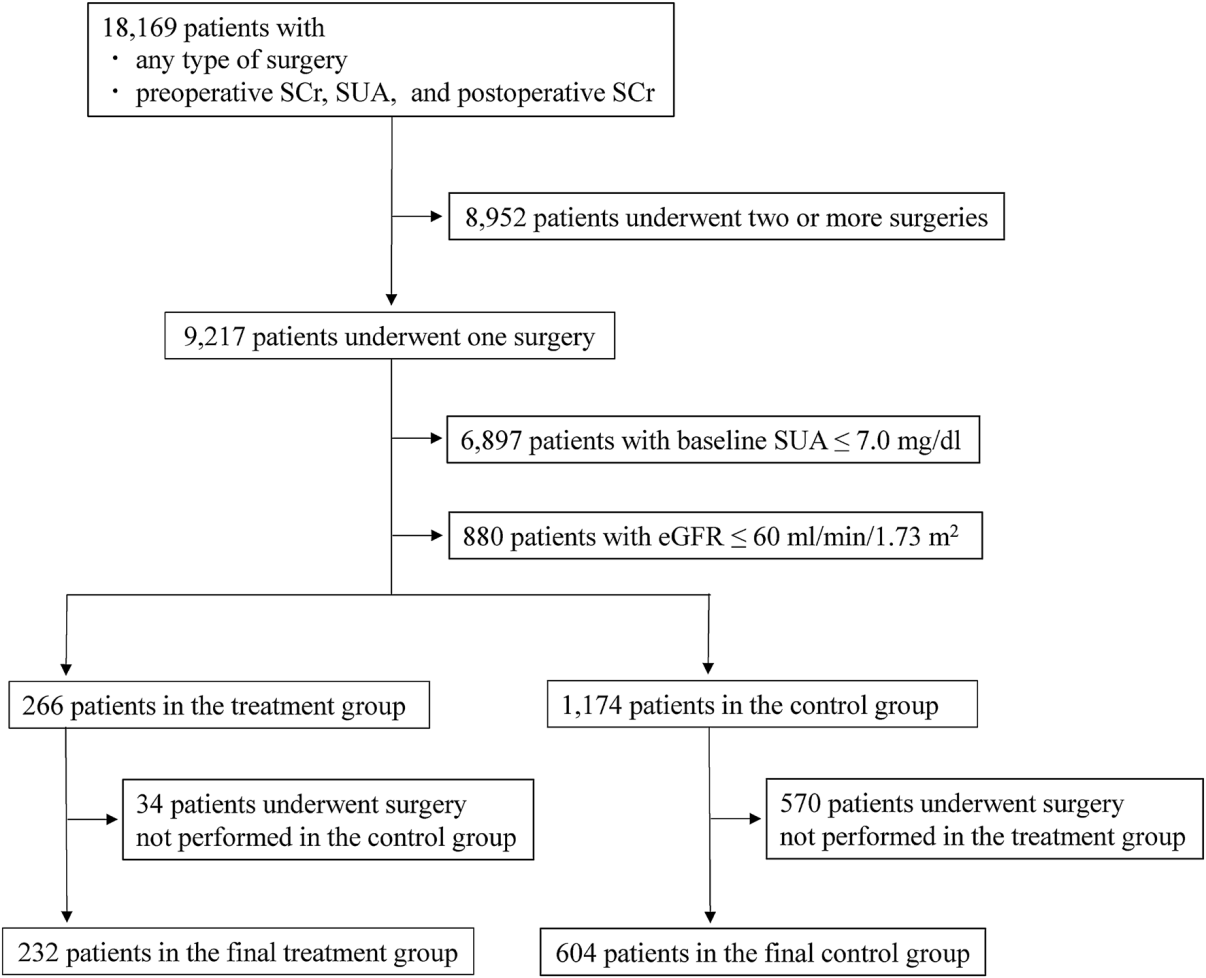
Flow chart of the study cohort selection. *SCr* serum creatinine, *SUA* serum uric acid, *eGFR* estimated glomerular filtration rates

The flow chart of the study population is shown in Fig. 1. The final study cohort included 836 patients, of whom 232 were in the treatment group and 604 were in the control group. There were 137 covariates, with 118 types of surgery common to both groups. In the treatment group, 13 patients underwent cardiovascular surgery, and 219 patients underwent non-cardiovascular surgery. In the control group, 41 patients underwent cardiovascular surgery, while 563 patients underwent non-cardiovascular surgery.

The patient characteristics in the treatment group, as well as the control group before and after adjustment, are shown in Table 1. After adjustment including baseline (pre-treatment) SUA levels, the incidence of pAKI in the treatment group (9.05%; 95% CI 6.04–12.1%) was significantly lower than that in the control group (14.2%; 95% CI 11.2–17.2%, Table 2). On the other hand, after adjusting for preoperative SUA levels (Table 2), there was no significant difference in the expected incidence of pAKI between the control group (10.2%; 95% CI 7.61–12.8%) and the treatment group (9.05%; 95% CI 7.27–10.8%).

**Table 1:** Demographic and clinical characteristics of treatment or control group before and after propensity score adjustment

	Treatment group (n = 232)	Unadjusted		Adjusted including baseline SUA		Adjusted including preoperative SUA	
		Control group (n = 604)	Absolute standardized difference	Control group	Absolute standardized difference	Control group	Absolute standardized difference
Age (years), mean ± SD	65.8 ± 14.0	61.4 ± 15.2	0.33	64.8 ± 13.7	0.069	66.4 ± 13.5	0.042
Male sex, %	91.8	87.9	0.13	89.4	0.082	90.1	0.059
eGFR (ml/min/1.73 m^2^), mean ± SD	82.5 ± 50.2	81.0 ± 36.3	0.03	80.8 ± 41.9	0.037	80.6 ± 28.4	0.036
Baseline (Pre-treatment) SUA (mg/dL), mean ± SD	9.19 ± 1.57	7.76 ± 0.88	1.12	8.92 ± 1.11	0.198		
Preoperative SUA (mg/dL), mean ± SD	5.47 ± 2.28		1.33			5.78 ± 1.61	0.157
Urinary lithiasis, %	3.4	1.5	0.13	2.8	0.04	3.6	0.01
Hypertension, %	65.5	39.4	0.54	58.0	0.16	61.7	0.08
Ischemic heart disease, %	34.5	18.9	0.36	32.1	0.05	35.8	0.03
Heart failure, %	35.8	19.4	0.37	30.1	0.12	31.9	0.08
Diabetes mellitus, %	37.9	25.0	0.28	35.5	0.05	36.2	0.04
Cancer, %	63.4	51.8	0.23	62.3	0.02	55.3	0.17
Liver disease, %	37.1	18.2	0.43	32.6	0.10	33.3	0.08
Diuretics, %	19.8	15.7	0.11	17.1	0.07	17.7	0.05
ACE inhibitors, %	9.9	4.4	0.21	9.2	0.02	8.1	0.06
ARBs, %	18.5	9.8	0.25	17.7	0.02	17.9	0.02
Antibiotics, %	37.1	31.5	0.12	34.3	0.06	33.8	0.07
NSAIDs, %	57.3	49.5	0.16	55.1	0.04	50.6	0.14
Anticancer drugs, %	1.7	3.0	0.08	1.5	0.07	1.2	0.04
Contrast media, %	9.1	8.8	0.01	8.9	0.01	9.0	0.003
Surgeries, %, mean [range]	0.8 [0.4–4.3]	0.8 [0.2–7.0]	0.06 [0–0.301]	0.8 [0.1–4.6]	0.04 [0–0.188]	0.8 [0.1–4.5]	0.04 [0–0.175]

*SD* standard deviation, *eGFR* estimated glomerular filtration rates, *SUA* serum uric acid, *ACE* angiotensin-converting enzyme, *ARBs* angiotensin II receptor blockers, *NSAIDs* nonsteroidal anti-inflammatory drugs

**Table 2:** Incidence of pAKI of treatment or control group before and after propensity score adjustment

	Treatment group (n = 232)	Unadjusted		Adjusted including baseline SUA		Adjusted including preoperative SUA	
		Control group (n = 604)	*p*	Control group	*p*	Control group	*p*
Incidence of pAKI, %	9.1	10.6	0.51	14.2	< 0.05	10.2	0.64

*pAKI* postoperative acute kidney injure, *SUA* serum uric acid

### Discussion

Our findings demonstrate, for the first time, that preoperative hyperuricemia treatment with allopurinol could prevent the development of pAKI in hyperuricemic subjects. Meanwhile, this preventive effect was not observed after adjusting the preoperative SUA levels. In line with this, previous studies have reported that SUA may have an important role in AKI development via multiple mechanisms, e.g., the intratubular deposition of uric acid crystals, renal vasoconstriction, and pro-inflammatory properties [17]. Taking together, these results indicate that decreased SUA levels before surgery secondary to allopurinol treatment, rather than allopurinol per se, may be associated with decreased incidence of pAKI.

In observational studies, selection bias usually arises due to non-randomized design. As shown in Table 2, the incidence of pAKI in the treatment group is comparable with the unadjusted incidence in control group. However, the rate of many confounding covariates are much higher in the treatment group than in the control group, i.e., 11 covariates had absolute standardized differences that exceeded 0.2. Hyperuricemia treatment is commonly based on the patient’s clinical characteristics, and thus the individuals in the treatment group may be in worse general condition. This may explain that there is no statistically significance in the incidence of pAKI between groups before adjustment. However, in this study, the propensity adjustment for non-equivalent comparison groups was successfully applied, i.e., all absolute standardized differences could achieve less than 0.2, and thus overcoming the potential bias.

In conclusion, our findings demonstrated that the preoperative hyperuricemia may be one of the modifiable risk factors, and thus pharmacological uric acid lowering before surgery could decrease the incidence of pAKI.

## Limitations

This study has several limitations. First, although the covariates were determined based on published studies, unknown factors could still bias the estimates. Especially, second, our analysis included only patients with preoperative normal renal function due to minimize the confounding factors. However, preoperative low eGFR is one of the most important predictors for pAKI development. Third, we used the clinical data from RYOMA 2, which provides longitudinal patient history allowing us to identify the subjects in the treatment or control group. However, RYOMA 2 does not include intraoperative information, such as anesthetic type, total bleeding volume, infusion balance, and blood transfusion. These factors are also well-known cofounding covariates in the development of pAKI. Furthermore, recent evidence demonstrates that dysfunction of ATP-binding cassette subfamily G member 2 (ABCG2), a high capacity urate exporter, is an important genetic risk factor in gout and hyperuricemia [18]. In contrast, several genetic mutations within a glucose and urate transporter gene were reported to cause renal hypouricemia [19]. The hereditary renal hypouricemia has been known to be relatively common in Japan, approximately 0.3%, and associated with reduced kidney function [20, 21]. Nevertheless, the link between these variants, as well as hypouricemia, and pAKI remains unknown. Therefore, further prospective investigation may be needed to address these clinically relevant questions in the future.

## Supplementary information

**Additional file 1.** Treatment details in the study population.

## Data Availability

All data generated or analysed during this study are included in this published article.

## Abbreviations

pAKI: postoperative acute kidney injury
RYOMA 2: Retrieval sYstem for Open Medical Analysis
SUA: serum uric acid
SCr: serum creatinine
eGFR: estimate glomerular filtration rate
KDIGO: Kidney Disease: Improving Global Outcomes
ACE: angiotensin-converting enzyme
ARBs: angiotensin II receptor blockers
NSAIDs: nonsteroidal anti-inflammatory drugs
SD: standard deviation
CI: confidence interval
ABCG2: ATP-binding cassette subfamily G member 2
