# Supplementary material for: Influence of hyperuricemia treatment on postoperative acute kidney injury among hyperuricemia patients: a single-center retrospective database analysis
Source: BMC Res Notes. 2019 Nov 21;12:756. doi: 10.1186/s13104-019-4783-1 (PMC6873755; doi:10.1186/s13104-019-4783-1)
Supplement: Supplementary file 1 — Additional file 1. Treatment details in the study population. [file 13104_2019_4783_MOESM1_ESM.docx]

**Additional Table S1.** Daily dose of allopurinol

| Doses | N | % |
| --- | --- | --- |
| ≤50 mg | 27 | 11.6% |
| 51-100 mg | 116 | 50.0% |
| 101-150 mg | 22 | 9.5% |
| 151-200 mg | 49 | 21.1% |
| ≥201 mg | 18 | 7.8% |

*N*; number of patients

**Additional Table S2.** Duration of allopurinol treatment

| Duration | N | % |
| --- | --- | --- |
| ≤30 days | 45 | 19.4% |
| 31-60 days | 36 | 15.5% |
| 61-90 days | 29 | 12.5% |
| 91-180 days | 19 | 8.2% |
| 181-270 days | 11 | 4.7% |
| 271-360 days | 5 | 2.2% |
| ≥361 days | 87 | 37.5% |

*N;* number of patient
